# Prevalence of hypertension among adolescents: systematic review and meta-analysis

**DOI:** 10.1590/S1518-8787.2016050006236

**Published:** 2016-05-06

**Authors:** Vivian Siqueira Santos Gonçalves, Taís Freire Galvão, Keitty Regina Cordeiro de Andrade, Eliane Said Dutra, Maria Natacha Toral Bertolin, Kenia Mara Baiocchi de Carvalho, Mauricio Gomes Pereira

**Affiliations:** I Programa de Pós-Graduação em Nutrição Humana. Faculdade de Ciências da Saúde. Universidade de Brasília. Brasília, DF, Brasil; II Hospital Universitário Getúlio Vargas. Universidade Federal do Amazonas. Manaus, AM, Brasil; III Programa de Pós-Graduação em Ciências Médicas. Faculdade de Medicina. Universidade de Brasília. Brasília, DF, Brasil

**Keywords:** Adolescent, Hypertension, epidemiology, Prevalence, Meta-Analysis

## Abstract

**OBJECTIVE:**

To estimate the prevalence of hypertension among adolescent Brazilian students.

**METHODS:**

A systematic review of school-based cross-sectional studies was conducted. The articles were searched in the databases MEDLINE, Embase, Scopus, LILACS, SciELO, Web of Science, CAPES thesis database and Trip Database. In addition, we examined the lists of references of relevant studies to identify potentially eligible articles. No restrictions regarding publication date, language, or status applied. The studies were selected by two independent evaluators, who also extracted the data and assessed the methodological quality following eight criteria related to sampling, measuring blood pressure, and presenting results. The meta-analysis was calculated using a random effects model and analyses were performed to investigate heterogeneity.

**RESULTS:**

We retrieved 1,577 articles from the search and included 22 in the review. The included articles corresponded to 14,115 adolescents, 51.2% (n = 7,230) female. We observed a variety of techniques, equipment, and references used. The prevalence of hypertension was 8.0% (95%CI 5.0–11.0; I^2^ = 97.6%), 9.3% (95%CI 5.6–13.6; I^2^ = 96.4%) in males and 6.5% (95%CI 4.2–9.1; I^2^ = 94.2%) in females. The meta-regression failed to identify the causes of the heterogeneity among studies.

**CONCLUSIONS:**

Despite the differences found in the methodologies of the included studies, the results of this systematic review indicate that hypertension is prevalent in the Brazilian adolescent school population. For future investigations, we suggest the standardization of techniques, equipment, and references, aiming at improving the methodological quality of the studies.

## RESUMO

**OBJETIVO:**

Estimar a prevalência de hipertensão arterial entre adolescentes escolares brasileiros.

**MÉTODOS:**

Foi realizada revisão sistemática de estudos transversais de base escolar. Os artigos foram pesquisados nas bases de dados Medline, Embase, Scopus, Lilacs, SciELO, *Web of Science*, Banco de teses da Capes e Tripdatabase. Além disso, foram examinadas as listas de referências bibliográficas dos estudos relevantes para identificar artigos potencialmente elegíveis. Não foram aplicadas restrições da data de publicação, idioma ou *status* de publicação. Os estudos foram selecionados por duas avaliadoras independentes que também extraíram os dados e avaliaram a qualidade metodológica seguindo oito critérios relacionados à amostragem, mensuração da pressão arterial e apresentação dos resultados. Calculou-se a metanálise utilizando modelo de efeitos randômicos e foram realizadas análises para investigação de heterogeneidade.

**RESULTADOS:**

Foram recuperados 1.577 artigos na busca, e 22 foram incluídos na revisão. Os artigos incluídos corresponderam a 14.115 adolescentes, sendo 51,2% (n = 7.230) do sexo feminino. Houve variedade de técnicas, equipamentos e referências utilizados. A prevalência de hipertensão foi 8,0% (IC95% 5,0–11,0; I^2^ = 97,6%), sendo no sexo masculino 9,3% (IC95% 5,6–13,6; I^2^ = 96,4%) e no feminino, 6,5% (IC95% 4,2–9,1; I^2^ = 94.2%). A metarregressão não identificou as causas da heterogeneidade entre os estudos.

**CONCLUSÕES:**

Apesar das diferenças encontradas nas metodologias dos estudos incluídos, os resultados desta revisão sistemática indicam que a hipertensão arterial é prevalente na população escolar adolescente no Brasil. Para investigações futuras sugere-se a padronização de técnicas, equipamentos e referências visando à melhoria da qualidade metodológica dos estudos.

## INTRODUCTION

Some risk factors for cardiovascular disease such as hypertension have been increasingly prevalent among adolescents and follow the growing trend of the cases of overweight, physical inactivity and inadequate nutrition in this population^32^ ^,^ ^a^ ^,^ ^b^.

Hypertension is a disease related to different causes, in which blood pressure levels remain high for a certain period. Organs such as the heart, brain, kidneys, and blood vessels are usually affected and undergo changes that may compromise their functions. This condition is also often related to metabolic changes and one of the most common risk factors for cardiovascular disease^42^ ^,^ ^43^. It is usually asymptomatic in adolescence, which hinders early diagnosis^3^. However, its detection, treatment and control are cornerstones to the reduction of cardiovascular events.

In a systematic review of studies with Brazilian adolescents conducted until 2008, without presence of the North region, the prevalence of hypertension was estimated at 8.0%^23^. For the adult population, the most recent nationwide studies usually obtain the prevalence of hypertension by self-reported medical diagnosis. In 2013, the *Vigilância de Fatores de Risco e Proteção para Doenças Crônicas por Inquérito Telefônico* (VIGITEL – Surveillance of Risk and Protection Factors for Chronic Diseases by Telephone Survey) system found a 24.1% prevalence in individuals older than 18 years, and the *Pesquisa Nacional de Saúde* (PNS – National Health Survey), 21.4%. Despite the methodological differences, these data reinforce the evolution of hypertension over the course of life, indicating the importance of monitoring and planning early and appropriate interventions^c^ ^,^ ^d^.

Brazil currently lacks a national survey investigating the prevalence of hypertension in all age groups of adolescence; thus, a systematic review with a meta-analysis on this theme can help overcome this gap, showing the prevalence of the disease and providing a base for planning and managing public policies related to adolescent health. In this context, our objective was systematically reviewing studies on the prevalence of hypertension in adolescent Brazilian students to estimate its magnitude and evolution.

## METHODS

### Protocol and registration

The protocol for the review was registered on the International Prospective Register of Systematic Reviews (PROSPERO) database under the number CRD42015016263.

### Eligibility criteria

We considered eligible the school-based cross-sectional studies conducted in Brazil evaluating the prevalence of hypertension in adolescents (aged 10 to 19 years). To be included, the studies needed to meet the following criteria: hypertension at or above the 95^th^ percentile for sex, height and age (10 to 17 years) or above 140 x 90 millimeters of mercury (mmHg) in adolescents aged 18 to 19 years^43^ and presence of public and private schools in the same study. Publication date, language or status limitations were not applied. We excluded studies assessing adolescents with specific conditions (hypertension or parents with hypertension, obesity, diabetes, chronic kidney disease, among others) and those with self-reported blood pressure.

### Information sources and search strategies

The following databases were searched: MEDLINE (via PubMed), Scopus, Embase, Web of Science, Adolec, Scientific Electronic Library Online (SciELO), Latin American and Caribbean Health Science Literature (LILACS), Trip Database, and the Brazilian Theses and Dissertation Database (via CAPES).

The search strategy for MEDLINE (via PubMed) was: (“Adolescent”[Mesh] OR “Adolescent”[TIAB] OR “Teenager”[TIAB] OR “Students”[Mesh] OR “Students”[TIAB] OR “Child”[Mesh] OR “Child” [TIAB]) AND (“Hypertension”[Mesh] OR “Hypertension”[TIAB] OR “Blood pressure”[Mesh] OR “Blood pressure”[TIAB] OR “Blood arterial”[TIAB]) AND (“Surveys”[TIAB] OR “Cross-Sectional Studies”[Mesh] OR “Cross-Sectional Studies”[TIAB] OR “Prevalence”[Mesh] OR “Prevalence”[TIAB] OR “Frequency”[TIAB]) AND (“Brazil”[Mesh] OR “Brazil”[TIAB] OR “Brasil”[TIAB]). This strategy was adapted for the other databases.

The searches started in September 2014 and the last one was in October 2014. In addition, the lists of references of relevant studies were examined to identify potentially eligible articles.

### Selection of studies and data extraction

According to the eligibility criteria, authors VSSG and KRCA selected studies independently over two stages, first evaluating the title and abstract and later reading the full text. Disagreements were resolved by consensus.

For data extraction, a spreadsheet was developed, including: title of the study, authors, year of data collection, publications arising from the study, city, state, objective, age group, existence of prior sample estimation, sample type and size, prevalence of hypertension, characteristics of measurement, method, reference, and equipment used.

Authors of articles lacking information were contacted at least twice for clarification.

### Assessment of the methodological quality of studies included

The instrument for critical appraisal of prevalence studies proposed by Loney et al.^21^ was used, with adaptations, to determine the quality of the articles. The authors adopted eight criteria: 1) census or probability sampling; 2) sampling source (official census, school census, among others); 3) previously estimated sample size; 4) proper measurement method (using validated equipment); 5) unbiased measurement performed by trained evaluators; 6) adequate response rate (> 70.0%) and description of refusals; 7) presentation of confidence intervals and analyses of subgroups of interest; and 8) study subjects well described and similar to the research question.

Studies received one point for each criterion met. Studies were considered of high quality if they scored 7 or 8 points; moderate quality, 4 to 6 points; and low quality, 0 to 3 points. Quality assessment was not used as a criterion for article exclusion, but as a parameter for the study of heterogeneity and analysis of subgroups.

### Data analysis

The primary outcome was the prevalence of arterial hypertension, with a 95% confidence interval (95%CI). Summary measures were estimated for the total population and subgroups according to sex, study quality, method and number of measurements, sample estimation, and region. The meta-analysis was estimated using a random effects model and weighted by the inverse of the variance, while heterogeneity was evaluated by the chi-squared test with a significance of p < 0.10 and its magnitude was measured by the I-squared (I^2^)^36^.

Meta-regressions were carried out to identify the causes of heterogeneity, using the test by Knapp and Hartung^19^ to test the following variables: quality score, sample size, ratio of female adolescents, year of the study, and number of blood pressure measurements. Small-study effect was assessed by funnel chart visual inspection and Egger’s test^45^.

Analyses were performed with the command “Metaprop” of the Stata software (version 12.0), adopting a significance of p < 0.05.

## RESULTS

**Figure 1:**
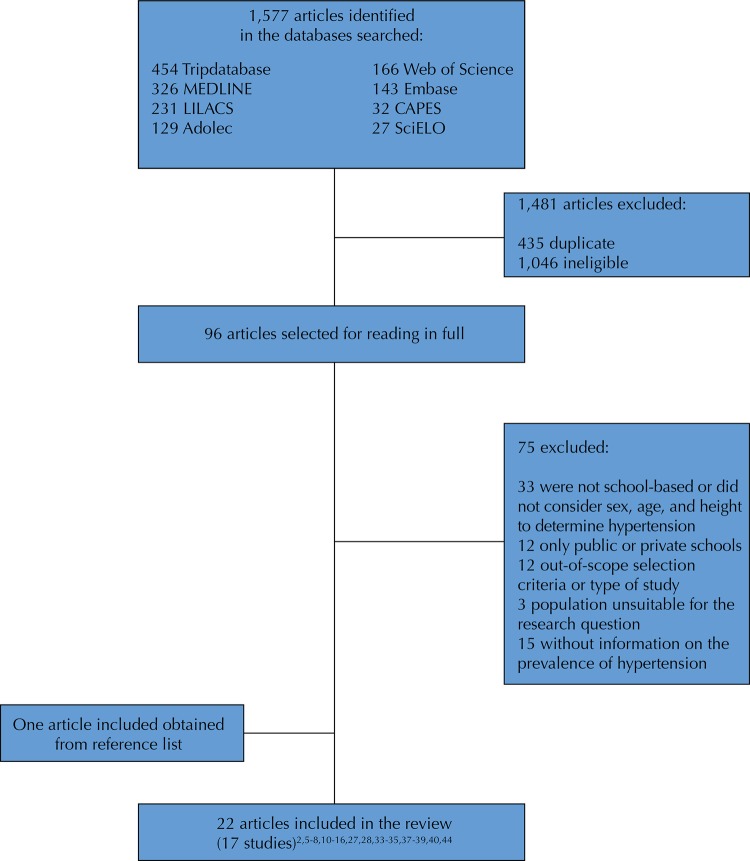
Flowchart of the search result, selection and inclusion of studies.

Figure 1 outlines the stages of selection of the studies and the final number of those eligible for the review, as well as the reasons for exclusion of studies. We received data from authors of eight studies^5^ ^,^ ^6^ ^,^ ^15^ ^,^ ^27^ ^,^ ^38^ ^,^ ^39^. After this, all studies had enough data to be included in the meta-analysis.

The studies included evaluated approximately 14,000 adolescents, most of them female. They represented all ages of adolescence and all Brazilian regions. The evaluations were carried out from 2000 to 2012. Other characteristics are summarized in Table 1.

**Table 1:** Characteristics of studies included in the systematic review on the prevalence of hypertension in adolescent Brazilian students.

Study	Research year	City, Federative Unit	Age group (years)	Sample size	Method of blood pressure measurement	Number of measurements	Reference or criterion used	Quality score
Almeida FA et al.^2^ (2011)	-	Sorocaba, SP	15-19	410	Auscultatory	3	SBC^42^ (2006); NHBPEP^30^ (2004)	4
Burgos MS et al.^5^ (2010)	2008	Santa Cruz do Sul, RS	10-17	1,251	Auscultatory	3	SBC^42^ (2006)	4
Burgos MS et al.^6^ (2013)	2005	Santa Cruz do Sul, RS	10-18	1,437	Auscultatory	3	SBC^43^ (2010)	5
Cândido AP et al.^7^ (2009)	2006	Ouro Preto, MG	10-14	487	Oscillometric	3	SBC^42^ (2006)	6
Christofaro DGD et al.^8,10,11,12^ (2009; 2011; 2013; 2014)	2008	Londrina, PR	10-17	1,021	Oscillometric	2	SBC^41^ (2005); NHBPEP^30^ (2004)	8
Cruz LL et al.^13^ (2013)	2010-2011	Alegre, ES	11-15	521	Auscultatory	3	SBC^43^ (2010)	4
Cureau FV et al.^14,15^ (2013;2014)	2011	Santa Maria, RS	14-19	1,072	Oscillometric	2	SBC^43^ (2010)	6
Faria ER et al.^16^ (2014)	2011-2012	Viçosa, MG	10-19	800	Oscillometric	3	SBC^43^ (2010)	6
Monego ET et al.^27^ (2006)	2001-2002	Goiania, GO (2011-2012)	10-14	2,118	Auscultatory	2	NHBPEP^29^ (1996)	5
Moura AA et al.^28^ (2004)	2000-2002	Maceio, AL	11-17	898	Auscultatory	2	NHBPEP^29^ (1996)	6
Polderman J et al.^33^ (2011); Roelofs et al.^37^ (2010)	2008	Aracaju, SE	12-17	1,002	Oscillometric	3	NHBPEP^30^ (2004)	7
Ribas AS et al.^34^ (2014)	2005	Belem, PA	10-19	420	Auscultatory	2	Koch VH^39^ (2000)	7
Rinaldi AEM et al.^35^ (2012)	2006-2007	Botucatu, SP	10-14	389	Auscultatory	3	NHBPEP^30^ (2004)	4
Rosa MLG et al.^38^ (2007)	2003-2004	Niteroi, RJ	12-17	456	Oscillometric	6	NHBPEP^30^ (2004)	8
Silva KS et al.^40^ (2009)	2005	Joao Pessoa, PB	10-12	705	Auscultatory	2	NHBPEP^30^ (2004)	6
Silva KSS et al.^36^ (2007)	2005	Joao Pessoa, PB	14-17	674	Auscultatory	2	NHBPEP^30^ (2004)	5
Stabelini Neto A et al.^44^ (2011)	2009	Curitiba, PR	10-17	454	Auscultatory	2	NHBPEP^30^ (2004)	7

SBC: *Sociedade Brasileira de Cardiologia* (Brazilian Society of Cardiology); NHBPEP: National High Blood Pressure Education Program; Koch VH: Vera H. Koch

Participants were selected by probability sampling, usually by clusters of two or more stages; the study by Rinaldi et al.^35^ was the exception. Studies used official census or school census data to estimate samples; however, five of them did not specify their source of population data for this stage^5^ ^,^ ^6^ ^,^ ^13^ ^,^ ^27^ ^,^ ^35^. The studies had a mean quality of 5.8 points and five were classified as high quality^8^ ^,^ ^10^ ^-^ ^12^ ^,^ ^33^ ^,^ ^34^ ^,^ ^37^ ^,^ ^38^ ^,^ ^44^.

Of the studies employing the auscultatory method for blood pressure measurement, three reported using a mercury column sphygmomanometer^2^ ^,^ ^5^ ^,^ ^28^, one failed to present this information^34^ and the others used an aneroid sphygmomanometer, an automatic equipment. The fourth report on the diagnosis, evaluation, and treatment of high blood pressure in children and adolescents^30^ was the methodological reference most often used to define hypertension (47.0%; n = 8).

Even for studies using the same reference, the number of measures was not standardized, varying between 2, 3, and 6, with most studies adopting the mean among them^2^ ^,^ ^8^ ^,^ ^16^ ^,^ ^33^ ^,^ ^38^ ^,^ ^39^ ^,^ ^44^. Three studies classified hypertension by the lowest value among measurements^5^ ^,^ ^6^ ^,^ ^40^ and the other studies defined it by the second measurement^27^ or the altered measurement^28^. One study failed to show this information^7^. Nine studies (53.0%) reported discarding outliers in the evaluation.

**Figure 2:**
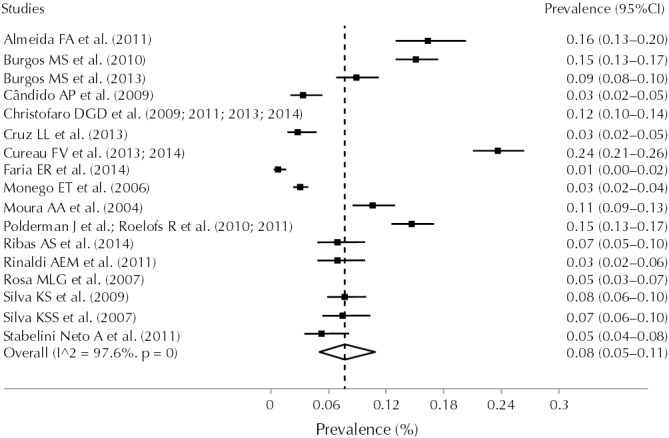
Prevalence of hypertension in Brazilian adolescents.

Ribas et al.^34^ did not stratify the prevalence of hypertension by sex and, therefore, this study could not be included in the analysis of subgroups of the meta-analysis. The estimated prevalence of hypertension for the entire population in the studies was 8.0% (95%CI 5.0–11.0; I^2^ = 97.6%) (Figure 2). The analysis by subgroups is shown in Table 2.

Prevalence (absolute frequency)

**Table 2:** Prevalence of hypertension, by subgroup, in Brazilian adolescents.

Subgroup	Number of studies	Total number of participants	Prevalence (%)	95%CI	I^2^ (%)	p (Chi-squared)
Sex						
Female	16	7,230	6.5	4.2–9.1	94.2	< 0.001
Male	16	6,887	9.3	5.6–13.6	96.9	< 0.001
Quality						
High	5	3,353	8.4	4.9–12.5	93.6	< 0.001
Moderate	12	10,762	7.5	4.1–11.6	98.2	< 0.001
Method of measurement						
Auscultatory	11	10,349	7.4	4.9–10.4	96.0	< 0.001
Oscillometric	6	3,766	8.2	2.6–16.5	98.7	< 0.001
Number of measurements						
Three or more	9	6,753	6.7	3.3–11.2	97.6	< 0.001
Two	8	7,362	8.9	4.8–14.1	98.0	< 0.001
Sample estimation						
Yes	12	9,556	6.0	3.7–8.8	96.4	< 0.001
No	5	4,559	12.6	6.9–19.7	97.6	< 0.001
Region						
North	1	420	6.9	4.7–9.5	-	-
Northeast	4	3,279	10.0	6.9–13.5	90.1	< 0.001
Midwest	1	2,118	3.0	2.3–3.8	-	-
Southeast	6	3,063	4.3	1.4–8.5	95.8	< 0.001
South	5	5,235	12.4	7.4–18.3	97.3	< 0.001

**Figure 3:**
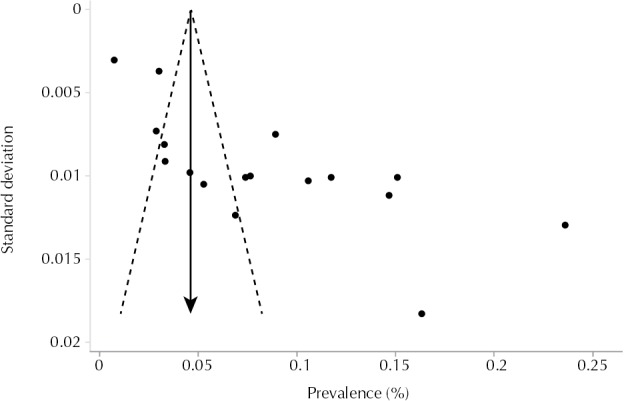
Funnel chart: prevalence of hypertension in each study by the standard deviation of the studies.

Figure 3 shows asymmetry between investigations, which was confirmed by Egger’s test (p < 0.001), indicating the possibility of small studies that probably found low prevalence not having been published.

Prevalence (absolute frequency); Standard deviation (absolute frequency)

In meta-regression, none of the analyzed variables was able to explain the causes for high heterogeneity among studies (p > 0.05).

## DISCUSSION

The evidence from cross-sectional studies indicated an estimated prevalence that emphasizes the importance of hypertension to public policy development, starting in adolescence. We also stress that the financial impact of the follow-up and intervention might be substantial even at this age^17^. The South region showed the highest prevalence and the Midwest, the smallest. Although we observed no evolution in prevalence from the last systematic review on this theme^23^, the results emphasize the importance of studies like this one, able to provide bases for health care and management in Brazil.

High heterogeneity was present in all the analyses, which requires caution in extrapolating the results, and the meta-regression was unable to explain the causes for that. However, studies with small sample sizes had an influence on this difference, which may have contributed to some bias in the results. Besides methodological causes, the great heterogeneity of the results may be due to natural differences among the subjects included in the studies, since states and cities are socioeconomically and culturally different from each other.

One of the potential limitations is the blood pressure measurement methods used in the original studies. Measurements vary significantly among the references and in relation to their adaptations and interpretations, which may influence the summarization of the prevalence. The most recent Brazilian recommendation stresses the importance of measuring in both arms and choosing the one presenting greater values for repetition, as well as excluding outliers and using the mean of the last two measurements. For diagnosis, this procedure should be repeated at different times^43^. None of the included studies reported using this complete methodology, which may have influenced the results directly, favoring the overestimation of prevalence. Studies indicate decreasing prevalence when blood pressure is measured repeatedly at different times^26^ ^,^ ^46^.

Differences in study scenarios and the lack of method standardization, reflected in different equipment, use of outliers when estimating the mean, and different intervals between measurements, may have contributed to heterogeneity. To mitigate this, we analyzed possible subgroups and used meta-regression. Nevertheless, the lack of explanation indicates that the study of subgroups should consider other subject characteristics such as nutritional status, age stratified by year, stages of adolescence and sexual maturity. However, the absence of this information in most original articles prevented the analysis.

We took measures to mitigate biases recurrent in studies of this nature. The methods of this work follow current recommendations for systematic reviews: sensitive search in the literature, without restrictions on language or publication date, research in grey literature, data selection, independent data extraction, and quality assessment of the studies. In addition, all included studies assessed public and private school students, seeking a greater representation of adolescent students. We also assessed the methodological quality of the studies and stratified statistical analysis by quality. We asked the authors about criteria not detailed in their articles.

While selecting studies evaluating the general population instead of the school population would have represented Brazilian adolescents better, we opted for the latter because few studies with this age group are conducted outside the school environment. In Brazil, in 2012, 97.4% of the population aged six to 14 years and 87.7% of the aged 15 to 19 years had access to school, regardless of monthly income classification^e^. Therefore, school is an important place for monitoring adolescent health^f^.

However, we consider the impossibility of measuring hypertension among adolescents who do not attend school as a study limitation, since this subgroup might change the profile found, with a possible underestimation of results.

Some previous studies also reviewed the literature on hypertension in adolescents. A systematic review with meta-analysis, with studies conducted in four Brazilian regions and data collected until 2008, showed similar results, with prevalence around 8.0% and higher for boys. The most and less prevalent regions were the South and Midwest, respectively. All subgroups examined in the study are also characterized by high heterogeneity^23^.

Comparing this earlier review with ours does not indicate an increase in the magnitude of the problem in this time span, but this must be interpreted carefully, because the features and quality of the studies included in each one differ in some aspects. The election of studies including only students from the public and private school networks was observed only in the current review. We aimed to improve the external validity and representativeness of all groups of the population of interest in the present review. Among the current studies, only one did not use probability sampling, while this feature was observed in five studies of the previous review. None of the current studies had samples with less than 250 adolescents, against four studies from the first review. The first review included a study in which blood pressure was measured only once, which did not occur in this review. The North region was represented only in this review.

The characteristics mentioned directly affect the internal and external validity of studies included. We also noticed an improvement in the methodological quality of the latest studies compared with the older ones, enabling the establishment of more stringent eligibility criteria, leading to increased reliability of the review results, even though we still observed lack of standardization. Thus, we cannot guarantee that hypertension in the adolescent school population has not increased in recent years, but we believe the current estimate represents it with smaller overestimation of the results.

Other three systematic reviews on this theme found great variation among rates, with 2.0% to 30.0%^22^ and 2.0% to 50.0%^4^ for Brazilian adolescents and 0.5% to 20.0% for representatives of America, Europe, Asia, and Africa^9^.

The reviews evaluated, as well as this work, indicate wide variations both in prevalence and in measurement procedures, besides selection criteria for subjects and conduction of the studies included.

In primary studies, with individuals of other nationalities, prevalence among Croatian (8.5%)^31^ and Portuguese (12.1%)^24^ adolescents was higher than among adolescent Brazilian students, while among Americans (3.0%)^25^, Egyptians (4.0%)^1^, and Hungarians (2.1%)^18^ it was lower.

## CONCLUSION

High blood pressure has a high prevalence in the adolescent population in Brazil. Future investigations need to standardize techniques and references, besides analyzing important factors for this population such as nutritional status, age, stages of sexual maturity and adolescence, to mitigate the high heterogeneity.

## Footnotes

a Instituto Brasileiro de Geografia e Estatística. Pesquisa de Orçamentos Familiares 2002-2003: antropometria e análise do estado nutricional de crianças e adolescentes no Brasil. Rio de Janeiro; 2006 [cited 2015 Oct 11]. Available from: http://ibge.gov.br/home/estatistica/populacao/condicaodevida/pof/2003medidas/default.shtm

b Instituto Brasileiro de Geografia e Estatística. Pesquisa de Orçamentos Familiares 2008-2009: antropometria e análise do estado nutricional de crianças, adolescentes e adultos no Brasil. Rio de Janeiro; 2010 [cited 2015 Oct 11]. Available from: http://www.ibge.gov.br/home/estatistica/populacao/condicaodevida/pof/2008_2009_encaa/

c Ministério da Saúde, Secretaria de Vigilância em Saúde. Vigitel Brasil 2013: vigilância de fatores de risco e proteção para doenças crônicas por inquérito telefônico. Brasília (DF); 2014 [cited 2015 Oct 11]. Available from: http://www.prefeitura.sp.gov.br/cidade/secretarias/upload/saude/arquivos/morbidade/Vigitel-2013.pdf

d Instituto Brasileiro de Geografia e Estatística. Pesquisa Nacional de Saúde 2013: percepção do estado de saúde, estilos de vida e doenças crônicas, Brasil, Grandes Regiões e Unidades da Federação. Rio de Janeiro; 2014 [cited 2015 Oct 11]. Available from: http://www.ibge.gov.br/home/estatistica/populacao/pns/2013/

e Instituto Brasileiro de Geografia e Estatística. Pesquisa Nacional por Amostra de Domicílio: síntese de indicadores 2011. Rio de Janeiro; 2012 [cited 2015 Oct 11]. Available from: http://www.ibge.gov.br/home/estatistica/populacao/trabalhoerendimento/pnad2011/default_sintese.shtm

f Instituto Brasileiro de Geografia e Estatística. Pesquisa Nacional de Saúde do Escolar 2012. Rio de Janeiro; 2013 [cited 2015 Oct 11]. Available from: http://www.ibge.gov.br/home/estatistica/populacao/pense/2012/
